# Analysis of VEGF-A Regulated Gene Expression in Endothelial Cells to Identify Genes Linked to Angiogenesis

**DOI:** 10.1371/journal.pone.0024887

**Published:** 2011-09-13

**Authors:** Corban G. Rivera, Sofie Mellberg, Lena Claesson-Welsh, Joel S. Bader, Aleksander S. Popel

**Affiliations:** 1 Department of Biomedical Engineering, Johns Hopkins University, Baltimore, Maryland, United States of America; 2 High-Throughput Biology Center, Johns Hopkins School of Medicine, Baltimore, Maryland, United States of America; 3 Department of Immunology, Genetics and Pathology, Uppsala University, Uppsala, Sweden; Virginia Commonwealth University, United States of America

## Abstract

Angiogenesis is important for many physiological processes, diseases, and also regenerative medicine. Therapies that inhibit the vascular endothelial growth factor (VEGF) pathway have been used in the clinic for cancer and macular degeneration. In cancer applications, these treatments suffer from a “tumor escape phenomenon” where alternative pathways are upregulated and angiogenesis continues. The redundancy of angiogenesis regulation indicates the need for additional studies and new drug targets. We aimed to (i) identify novel and missing angiogenesis annotations and (ii) verify their significance to angiogenesis. To achieve these goals, we integrated the human interactome with known angiogenesis-annotated proteins to identify a set of 202 angiogenesis-associated proteins. Across endothelial cell lines, we found that a significant fraction of these proteins had highly perturbed gene expression during angiogenesis. After treatment with VEGF-A, we found increasing expression of HIF-1α, APP, HIV-1 tat interactive protein 2, and MEF2C, while endoglin, liprin β1 and HIF-2α had decreasing expression across three endothelial cell lines. The analysis showed differential regulation of HIF-1α and HIF-2α. The data also provided additional evidence for the role of endothelial cells in Alzheimer's disease.

## Introduction

Angiogenesis has been implicated in a wide spectrum of diseases including cancer, age-related macular degeneration, rheumatoid arthritis, diabetic nephropathy, pathologic obesity, and asthma. Compounds that inhibit angiogenesis represent potential therapeutics for many diseases. Judah Folkman performed pioneering research in the field of angiogenesis [1]; his work led to the identification of a number of proteins and polypeptides with anti-angiogenic activity [2]. Since then, many compounds have entered clinical trials as modulators of angiogenesis. Targeting the vascular endothelial growth factor (VEGF) pathway has been the leading anti-angiogenic strategy in the clinic [3]. Other anti-angiogenic targets include integrins [4] and angiopoietin 1 [5]. While these therapies suppress new blood vessel growth for a period of time, anti-angiogenic therapies suffer from the upregulation of compensating pathways that circumvent the inhibited pathway. Consequently, the field is in need of a comprehensive understanding of the proteins and pathways involved in angiogenesis.

Identifying angiogenesis-associated proteins is related to the problem of gene function prediction. Over the past decade, there have been many important studies on using network structure to functionally annotate gene products. These methods were reviewed in [6], [7]. Early methods transferred gene functions from direct neighbors to annotate genes. Extensions to these methods allowed more distant annotations through shortest paths [8] and diffusion [9], [10], [11], [12]. Some of these methods make use of diverse machine learning techniques including SVMs [9] and Bayesian networks [10].

There has been recent interest in the area of applying bioinformatics approaches to study angiogenesis. These studies integrate gene expression data with molecular interactions with diverse goals. A recent study explored pathways associated with VEGF [11]. Another study validated their new method by recovering pathways known to be involved in angiogenesis [12]. A third study aimed to predict the impact of anti-angiogenic kinase inhibitors [13]. A previous study conducted in our lab identified crosstalk between angiogenesis modulating protein families [14]. These studies suggest momentum towards using bioinformatics to study angiogenesis. However none of these studies had the goal of finding new and missing angiogenesis gene annotations. We aimed to: (i) identify novel and missing angiogenesis annotations and (ii) verify their association with angiogenesis with statistical analysis and multiple gene expression datasets.

## Results and Discussion

**Figure 1:**
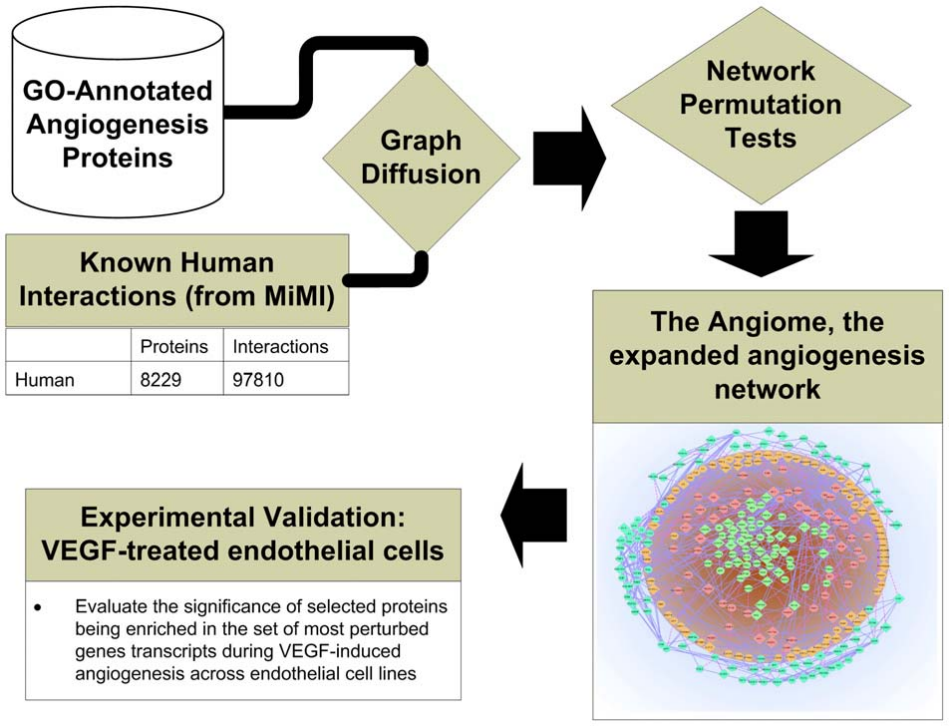
Computational strategy to identify and verify novel angiogenesis-associated proteins. We began by integrating known angiogenesis-annotated proteins (GO-annotated) with a set of known human interactions (from MiMI). We used a graph diffusion approach to identify new angiogenesis-associated proteins using a “guilt-by-association” approach. We verified the set of selected genes using two approaches. First, we evaluated the statistical significance of the selected genes by repeatedly permuting the human interactome (statistical hypothesis given in Methods). Secondly, we integrated a time series gene expression experimental dataset obtained during VEGF-induced angiogenesis to determine if the selected genes are enriched in the set of most perturbed transcripts.

In Figure 1, we describe our computational strategy to identify and validate novel angiogenesis-associated proteins. To ensure that the first objective is satisfied, the basis for our search began with proteins known to be annotated with angiogenesis from the Gene Ontology database (GO). We integrated molecular interactions with the angiogenesis-annotated proteins to identify new highly associated proteins. We satisfied the second objective with statistical analysis of three published datasets (see Table 1) that showed the newly associated proteins frequently had perturbed gene expression during angiogenesis. We combined the angiogenesis annotated and associated sets of proteins to form the *Angiome*, the network of protein interactions associated with angiogenesis.

**Table 1:** VEGF stimulated time series gene expression datasets analyzed.

Ref.	Source	Cells	Culture	Timepoints
[15]	GDS3567	HUVEC	Gelatine	0 m, 30 m, 1 h, 2.5 h
[16]	GDS2039	HMVEC	Matrigel	30 m, 2 h, 2 h, 4 h, 8 h
[17]	Mellberg et al.	TIME	Collagen I	15 m, 1 h, 3 h, 6 h, 8 h, 12 h, 18 h, 24 h

Each dataset measured VEGF induced changes in endothelial cell gene expression over a time course. The datasets used different cell lines, cell culture, and time points for their studies.

### Angiogenesis annotation by graph diffusion

**Figure 2:**
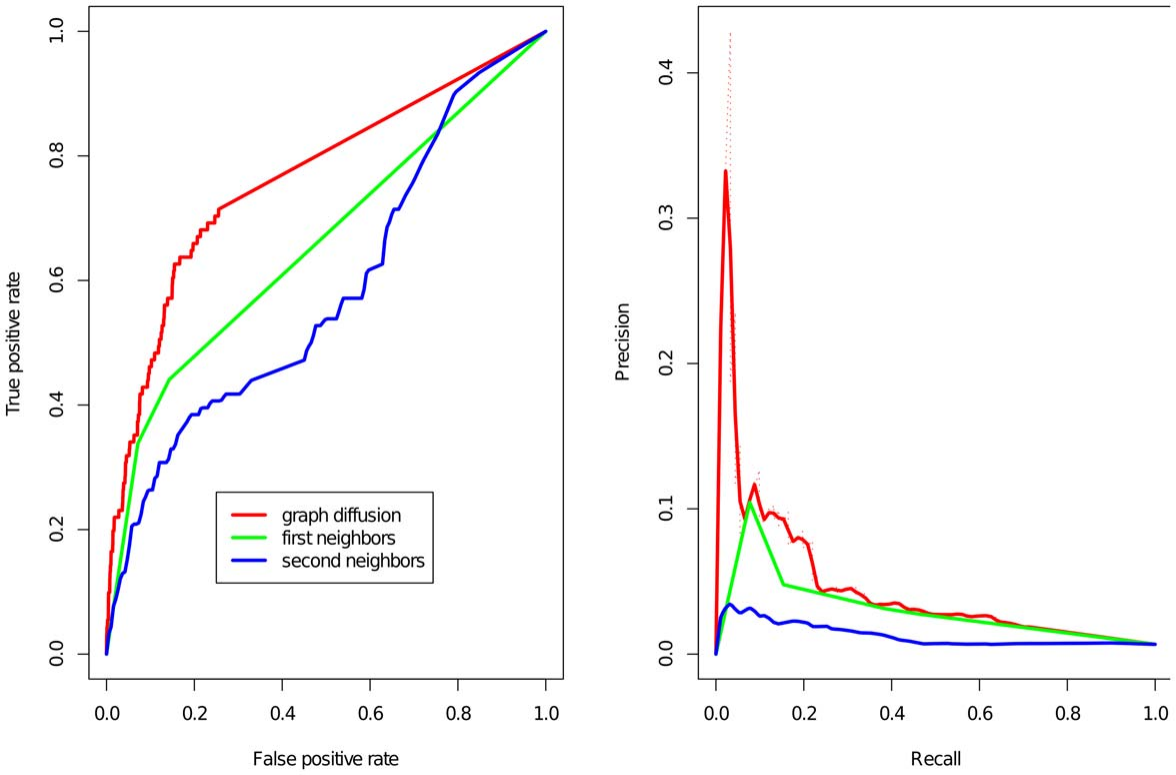
Graph diffusion for angiogenesis annotation. The receiver operator characteristic (ROC) curve and precision recall curve detailing the leave-one-out cross validation prediction accuracy of angiogenesis annotation. Using graph diffusion the area under the curve (AUC) was 0.76. Annotation by the first neighbors method achieved 0.66, and second neighbors method achieved 0.58.

To determine the quality of graph diffusion annotations, we compared graph diffusion to several basic methods for transferring functional annotations including a first neighbors method and a second neighbours method. Graph diffusion (see Methods) works by ranking proteins by their interactions with annotated proteins over paths of all lengths. The accumulation of evidence from all path lengths generally leads to better performance. The first neighbors method ranks proteins by the number of direct interactions with annotated proteins. The second neighbors method ranks proteins by the number of annotated proteins reachable by paths of length less than three. To evaluate the performance of these methods for angiogenesis annotations, we performed a leave-one-out cross-validation (LOOCV) procedure. In Figure 2, we show the performance of the diffusion kernel for angiogenesis annotation. The figure shows the receiver operator characteristic (ROC) curve and a precision recall curve for all methods. The performance of the methods can be summarized by the area under the ROC curve (AUC). Greater AUC indicates better performance. Graph diffusion achieves an AUC of 0.76, while the first and second neighbor methods achieved an AUC of 0.66 and 0.58 respectively. Based on this data, we proceeded to make angiogenesis annotations using graph diffusion.

### Validation by network randomization

The highest ranking proteins by graph diffusion are topologically closer to the annotated proteins; however the method can be biased towards hubs and other highly connected proteins. To eliminate this bias, we tested the statistical significance of a protein achieving as high of an association score in randomized networks. For a given protein, we test the null hypothesis that the score is equal to the rank that could be achieved in a randomized network with identical degree distribution. We generated 300 randomized networks by repeated edge swapping. The probability of the null hypothesis is given by the fraction of randomized networks where the score of the protein exceeds the score from the real network. We reject the null hypothesis if the probability is less than 0.01. Using this procedure, we identified a set of 202 angiogenesis associated proteins.

### The Angiome

**Figure 3:**
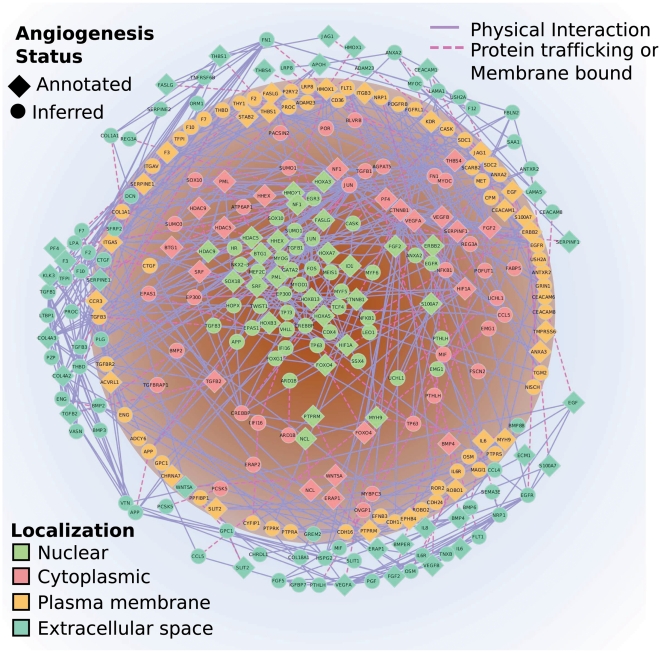
The Angiome. This figure represents all proteins currently annotated (diamond nodes) and topologically associated (circle nodes) with angiogenesis. The figure showed these proteins by their general cellular compartments: nucleus (green), cytoplasm (red), plasma membrane (orange), and extracellular (cyan). Protein trafficking between cellular components was shown as purple dashed lines. Protein-protein interactions within a compartment were shown as blue lines. The figure included the proteins in the single largest component of the network. A complete list of the proteins, interactions, and cellular component assignments could be found in the supplement.

The combined set included 293 angiogenesis annotated and associated proteins. The interactions (Table S1) and proteins (Table S2) included in this network are given as Supporting Information. In Figure 3, we visualized the combined network of angiogenesis-associated proteins (we call it the *Angiome*) by cellular compartment. Proteins were placed into the nucleus (green nodes), cytoplasm (yellow nodes), plasma membrane (orange nodes), or extracellular space (cyan nodes). Proteins that have been identified in multiple compartments were duplicated in the layout. Protein trafficking between compartments was indicated by dashed purple lines. Molecular interactions within a compartment were shown in grey. The interaction density of the figure illustrated the redundancy and complexity of the angiogenesis process.

### Validation by transcriptional profiling

Using graph diffusion, we identified 202 proteins that are significantly connected to angiogenesis-annotated proteins. We needed additional validation that these proteins were functionally related to angiogenesis. We expected that many angiogenesis-related genes would have VEGF-regulated gene expression in time during angiogenesis. We analysed three separate time series gene expression studies of angiogenesis involving VEGF stimulated endothelial cells. To mitigate potential cell line bias, we chose studies performed on HUVEC (GDS3567) [15], HMVEC (GDS2039) [16], and TIME [17] cells (see Table 1).

To identify transcripts with high temporal correlation after VEGF stimulation, we ranked transcripts by the absolute value of the covariance between the transcript measurements and the measured time points. For each protein, the temporal correlation of the gene expression profile was given as Supporting Information (Table S3). We tested the null hypothesis that the distribution of angiogenesis-associated proteins was uniform throughout the ranked list of transcripts. We determined the significance of the enrichment using the Kolmogorov-Smirnov test [18]. A significant *p*-value indicated that the angiogenesis-associated proteins were disproportionally ranked at either the head or tail of the ranked list of transcripts.

We first tested the set of angiogenesis-annotated genes (GO-annotated) to ensure that they were enriched in the ranked list of perturbed transcripts. As expected, we found that the set of angiogenesis-annotated (i.e. GO-annotated) proteins were significantly perturbed in HUVEC (*p* = 0.04) and TIME (*p* = 0.041) cell lines (see Table 2). We then tested the combined set of 293 proteins including the proteins found using graph diffusion and significant at the 0.01 level. We found that the combined set of proteins was more significantly perturbed than the set of GO-annotated proteins alone across all three endothelial cell lines (shown in Table 2).

**Table 2:** Enrichment of angiogenesis-associated proteins in a ranked list of the most perturbed gene expression transcripts during angiogenesis.

Gene Set	HUVEC	HMVEC	TIME
GO angiogenesis annotated only	0.04	0.185	0.041
angiogenesis annotated and associated at *p*<0.01	0.008	0.001	0.001
angiogenesis associated at *p*<0.01 only	0.08	0.001	0.001
angiogenesis annotated and associated at *p*<0.05	0.005	0.02	0.001
angiogenesis associated at *p*<0.05 only	0.035	0.026	0.008

To evaluate the relevance of angiogenesis-associated proteins with angiogenesis, we evaluated the significance of gene sets in the ranked list of most perturbed gene expression profiles. We obtained the gene expression profiles from three studies performed on different endothelial cell lines. The table gave the *p*-value of the enrichment of the gene set at the head of the ranked list of perturbed gene transcript.

### Genes with VEGF-regulated expression

We identified seven genes that were consistently regulated by VEGF-A across all three cell lines (shown in Table 3). Only genes with reliable gene expression measurements in all three datasets were considered. Genes such as HIV-1 tat interactive protein 2 (HTATIP2), HIF-1α, β-amyloid precursor protein (APP), and myocyte enhancer factor 2C (MEF2C) all had increasing gene expression across all three endothelial cell lines. Genes such as endoglin (ENG), liberin β1 (PPFIBP1), and EPAS1 (HIF-2α) had decreasing expression after VEGF treatment. We identified literature that supported the association of some of these proteins with angiogenesis. It was previously reported that HTATIP2 (also known as CC3) had antiangiogenic properties [19], and VEGF induced MEF2C expression [20]. We found that while endoglin had a known role in angiogenesis [21], the association between liperin β1and angiogenesis was not well established. Of these seven genes, only two had non-electronic angiogenesis annotations.

**Table 3:** Summary of genes temporally regulated by VEGF across cell lines.

Symbol	Name	Annotated	HMVEC	HUVEC	TIME
HTATIP2	HIV-1 Tat interactive protein 2	yes	0.72	0.71	0.86
HIF1A	hypoxia inducible factor 1	yes	0.59	0.85	0.89
APP	amyloid beta (A4) precursor protein	no	0.85	0.81	0.85
MEF2C	myocyte enhancer factor 2C	no	0.99	0.82	0.63
ENG	endoglin	no	−0.60	−0.62	−0.74
PPFIBP1	liprin beta 1	no	−0.61	−0.89	−0.79
EPAS1	endothelial PAS domain protein 1	no	−0.81	−0.93	−0.53

We highlighted those proteins from Figure 3 that had consistently correlated gene expression in time after VEGF stimulation. HTATIP2, HIF-1α, APP, and MEF2C had positively regulated gene expression, while ENG, PPFIBP1, and EPAS1 had negatively regulated gene expression. Correlation values are shown for HUVEC HMVEC and TIME endothelial cells. Only those genes with reliable measurements in all three datasets were considered.

It was known that HIF-1α was a transcription factor for VEGF [22]. Across three endothelial cell lines, we found a VEGF-regulated increase in HIF-1α expression over time. It was also established that HIF2A was a hypoxia responsive transcription factor [23]. We found that EPAS1 (also known as HIF-2α) had decreasing expression after VEGF-treatment across three endothelial cell lines. The analysis showed differential regulation of HIF-1α and HIF-2α.

It was speculated that endothelial cells may have a role in Alzheimer's disease [24], [25]. In this proposed mechanism, hypoxic brain cells produced VEGF and stimulated endothelial cells. It was also known that the disease resulted in the destruction of neurons by the accumulation of β-amyloid plaques (i.e. APP protein aggregates). After treatment with VEGF-A, we found increasing expression of APP across all three endothelial cell lines. These data supported the relation between endothelial cells and Alzheimer's disease.

### Pathways linked to angiogenesis

Using the expanded network shown in Figure 3, we identified overrepresented pathways linked to angiogenesis (see Table 4). The pathways we report in Table 4 could not be identified using angiogenesis-annotated proteins alone. A pathway associated with acute myocardial infarction (AMI) was identified as angiogenesis-associated with the expansion of the network to type IV collagens (i.e. COL4A3 and COL4A2) and coagulation factors (e.g. F10, F2, and F7). It was known that angiogenesis was a natural mechanism to restore perfusion after AMI [26]. The coagulation factors were also associated with prothrombin activation. Fragments of prothrombin had been identified as regulators of angiogenesis [27]. Collagen IV proteins also contributed to the platelet β-amyloid precursor protein pathway (APP). While the molecular basis was unclear, angiogenesis was identified as part of a mechanism for Alzheimer's disease along with pathological secretion of β-amyloid proteins [24]. In this study, the expansion of the angiogenesis-associated network gave us increased statistical power to link pathways with angiogenesis.

**Table 4:** Pathways significantly represented in the Angiome.

Pathway	Description	p-value
AMI	Acute myocardial infarction-associated pathway	2.00E-06
Extrinsic	Extrinsic prothrombin activation pathway	2.02E-04
Platelet APP	Platelet amyloid precursor protein pathway	3.50E-04
TGFB	TGF beta signaling pathway	3.00E-03
Nkt	Expression of chemokine receptors during T-cell polarization	3.80E-02
Keratinocyte	Keratinocyte differentiation	5.39E-02
Cardiac EGF	EGF receptor transactivation by GPCRs in cardiac hypertrophy	7.92E-02

These pathways could not be statistically identified from angiogenesis-annotated proteins alone. With the expansion of the angiogenesis-related network, these pathways were easily detected. The *p*-value was computed using Fisher's exact test followed by correction for the false discovery rate.

### Conclusions

Compounds that inhibit angiogenesis represent potential therapeutics for many diseases. Therapies that target the VEGF pathway have been used in the clinic. These treatments suffer from an “escape phenomenon” where alternative pathways are upregulated and angiogenesis continues. The redundancy of angiogenesis regulation indicates the need for additional studies and new drug targets.

To discover novel and missing angiogenesis annotations, we integrated the human interactome with known angiogenesis-annotated proteins to identify a set of 293 angiogenesis-related proteins. We verified these protein associations by repeated network randomization and analysis of relevant time series gene expression datasets. After treatment with VEGF-A, we found increasing expression of HIF-1α, APP, HIV-1 tat interactive protein 2, and MEF2C, while endoglin, liprin β1 and HIF-2α had decreasing expression across three endothelial cell lines. The analysis showed differential regulation of HIF-1α and HIF-2α. The data also provided additional evidence for the role of endothelial cells in Alzheimer's disease.

## Materials and Methods

The interaction dataset was taken from the Michigan Molecular Interaction database (MiMI) [28] (Feb 2009 version). The dataset was composed of 13,491 genes, proteins, and RNA connected by 126,763 physical interactions. The interactions included protein-protein, protein-DNA, protein-RNA, and RNA-RNA. As a result, the dataset captured diverse aspects of biomolecular interactions including protein complexation, transcriptional regulation, and RNA interference. The dataset consisted of interactions curated from reputable online databases such as Reactome [29], BIND, BioGrid [30], HPRD [31]. This network of physical interactions formed the basis for pathway expansion. Gene Ontology (GO) [32] annotations were used to determine the known set of angiogenesis-associated proteins (6/2010 version). MsigDB C2 was used for pathway annotations [33]. Preparation and computational preprocessing of the gene expression measurements was described in their manuscript.

### Graph diffusion

We used graph diffusion to establish topological associations. Intuitively, the algorithm worked by initiating a series of fixed length random walks through the network originating at nodes of interest. The score of each node was given by the fraction of random walks that pass through the node. A parameter γ controlled the length of the random walks. The aggregation of evidence over multiple paths led to a more stable result from potentially unreliable data. The steady-state solution of this system associated each node with an association score. In this study, we set γ such that all nodes receive some non-zero association score. We made this functionality available through our webserver (sysbio.bme.jhu.edu).

For a weighted undirected graph *G*(*V*,*E*) with vertex set *V* and edge set *E*, let **A** be the symmetric adjacency matrix representing *G*. Let *q_i_* be 1 if node *i* was in the query set or zero otherwise. Let *i∼j* be the set of nodes connected to node *i*. We expressed the time derivative 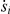 of the diffusion kernel score *s_i_*(*q*) for node 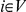 as
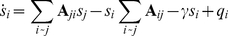
(1)


Let **D** be the degree weighted diagonal matrix of **A**. In matrix notation, equation (1)had the form in equation (2).
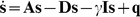
(2)


Our goal was to identify the values of **s** at steady-state. We set 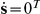 from equation (2) and solve for **s** in equation (3).
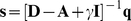
(3)


The values in **s** gave the association of each node with the query nodes defined by **q**.

### Statistical significance by network randomization

We computed the statistical significance of protein associations by permutation testing. We tested the null hypothesis that the association score of a protein is equal to the score of the protein in randomized networks. The alternative hypothesis was that the score of a protein is greater than the score of the protein in randomized networks. To test these hypotheses, we generated 300 randomly edge swapped networks. The probability of the null hypothesis was given by the number of randomized networks where the score of a protein exceeded the score of the protein in the real network. Pseudocounts were added to avoid fitted probabilities of zero. We did not evaluate the statistical significance of the angiogenesis-annotated proteins. Angiogenesis-annotated proteins were selected for the study and as such they are inherently biased.

### Time series gene expression analysis

We reanalyzed three time series gene expression datasets to validate the angiogenesis-associated proteins and mitigate potential cell line bias. In Table 1, we presented the three time series datasets used in this study. These studies focused on early transcriptional changes during VEGF induced angiogenesis. The studies used different endothelial cell lines and measured different time points. Mellberg *et al*. [17] measured gene expression of VEGF stimulated telomerase-immortalized human microvascular endothelial (TIME) cells at 15 min, 1, 3, 6, 9, 12, 18, and 24 hours. Schweighofer *et al.* [15] measured gene expression in VEGF simulated HUVEC at 30 min, 1, 2.5, and 6 hours (GDS3567). Glesne *et al*. [16] measured gene expression in VEGF stimulated HMVEC at 30 min, 1, 2, 4, and 8 hours of tubulogenesis (GDS2039).

To identify temporal correlations, we computed the Pearson's correlation coefficient and covariance between each gene expression profile and the time course. Genes with fewer than 3 reliable gene expression measurements are excluded. We formed a ranked list of perturbed genes using the absolute value of the covariance. We tested the null hypothesis that angiogenesis-annotated genes are evenly distributed throughout the ranked list of genes. The alternative hypothesis was that the proteins were disproportionally ranked at either the head or tail of the list. We used the gene set enrichment analysis (GSEA) software to compute the *p*-value [34]. Pathway enrichment was computed using Fisher's exact test followed by correction for the false discovery rate (i.e. Benjamini-Hochberg [35]).

## Supporting Information

Table S1 **Molecular interactions and types included in** **Figure 3** **.** The file listed the interactions included in Figure 3. Proteins trafficking between components and transmembrane proteins may be represented in multiple compartments.
(XLS)

Table S2 **Graph diffusion scores and associated p-values.** The data included the individual genes included in Figure 3. The file indicated if the protein was previously annotated with angiogenesis, the graph diffusion score, and the permutation-based *p*-value. Pseudocounts may have been added to avoid fitted probabilities of zero.
(XLS)

Table S3 **VEGF-induced gene expression correlation measurements.** For each protein, we list the temporal correlation of the gene expression profile during VEGF-induced angiogenesis. A positive (negative) correlation value indicated a trend towards increasing (decreasing) gene expression after VEGF treatment. Results were shown for the three VEGF-A induced gene expression datasets.
(XLS)
